# A new experimental design to study inflammation-related versus non-inflammation-related depression in mice

**DOI:** 10.1186/s12974-021-02330-9

**Published:** 2021-12-11

**Authors:** Pierre Cardinal, Camille Monchaux de Oliveira, Julie Sauvant, Aline Foury, Muriel Darnaudéry, Sylvie Vancassel, Nathalie Castanon, Lucile Capuron

**Affiliations:** 1 INRAE 1286, Nutrition and Integrative Neurobiology (NutriNeuro), UMR 1286, Bordeaux, France; 2 Bordeaux University, Nutrition and Integrative Neurobiology (NutriNeuro), UMR 1286, Bordeaux, France

**Keywords:** Depression, Inflammation, Stress, Obesity, Enzymatic pathways, Kynurenine, Tetrahydrobiopterin, Preclinical models

## Abstract

**Background:**

Major depressive disorder (MDD) represents a major public health concern, particularly due to its steadily rising prevalence and the poor responsiveness to standard antidepressants notably in patients afflicted with chronic inflammatory conditions, such as obesity. This highlights the need to improve current therapeutic strategies, including by targeting inflammation based on its role in the pathophysiology and treatment responsiveness of MDD. Nevertheless, dissecting the relative contribution of inflammation in the development and treatment of MDD remains a major issue, further complicated by the lack of preclinical depression models suitable to experimentally dissociate inflammation-related *vs.* inflammation-unrelated depression.

**Methods:**

While current models usually focus on one particular MDD risk factor, we compared in male C57BL/6J mice the behavioral, inflammatory and neurobiological impact of chronic exposure to high-fat diet (HFD), a procedure known to induce inflammation-related depressive-like behaviors, and unpredictable chronic mild stress (UCMS), a stress-induced depression model notably renowned for its responsivity to antidepressants.

**Results:**

While both paradigms induced neurovegetative, depressive-like and anxiety-like behaviors, inflammation and downstream neurobiological pathways contributing to inflammation-driven depression were specifically activated in HFD mice, as revealed by increased circulating levels of inflammatory factors, as well as brain expression of microglial activation markers and enzymes from the kynurenine and tetrahydrobiopterin (BH4) pathways. In addition, serotoninergic and dopaminergic systems were differentially impacted, depending on the experimental condition.

**Conclusions:**

These data validate an experimental design suitable to deeply study the mechanisms underlying inflammation-driven depression comparatively to non-inflammatory depression. This design could help to better understand the pathophysiology of treatment resistant depression.

**Supplementary Information:**

The online version contains supplementary material available at 10.1186/s12974-021-02330-9.

## Background

Major depressive disorder (MDD) is one of the leading cause of disabilities worldwide and a major health concern in modern societies. Despite the range of treatment options, many patients experience chronic relapse of the disease and one-third of them do not respond to conventional antidepressants [1]. To worsen the picture, MDD prevalence is steadily rising, notably in patients with chronic medical conditions associated with low-grade inflammation, including cardiovascular diseases, autoimmune diseases, metabolic disorders and obesity [2–5]. Importantly, these patients also often display increased resistance to antidepressants, as compared to those free from these comorbid conditions [6–8]. This alarming issue highlights the need for a better understanding of the pathophysiology of treatment resistant depression (TRD) and the identification of reliable phenotypic markers to characterize concerned patients, who represent a highly heterogeneous population.

TRD is likely a multidimensional condition, but recent evidence suggests the involvement of inflammatory processes [9, 10], in line with their notorious role in the pathophysiology of MDD [2, 11]. Enhanced baseline circulating levels of inflammatory markers predict poor antidepressant outcomes in depressed patients [9, 10, 12, 13]. Moreover, obesity-related inflammation, which is known to contribute to depressive comorbidity in obese subjects [3, 5, 14, 15], was recently found to also compromise response to standard antidepressants [6–8]. These findings sparked interest in the possibility of targeting inflammation to improve this clinical response [16–19]. The first studies conducted on this topic have provided promising results, although they vary depending on the class of anti-inflammatory drugs tested, their respective mechanism of action and potential neuromodulatory properties. Importantly, results also differ based on the clinical profile of depressed patients, with those with elevated inflammatory markers and poor response to antidepressants exhibiting greater benefit from anti-inflammatory interventions. Determining circulating levels of particular inflammatory markers in depressed patients was found to be useful for predicting responsiveness to regular antidepressants [16, 18, 20]. Nevertheless, deeply understanding the relative contribution of inflammatory processes to the induction and treatment of different depressive symptom dimensions in these patients is needed to move toward more tailored and personalized anti-inflammatory therapeutic strategies. Addressing this challenging issue has been so far complicated, particularly due to the lack of relevant and reliable animal models of depression, i.e., models allowing experimentally dissociating inflammation-related vs. inflammation-unrelated depressive-like behaviors.

Exposure to psychological and/or environmental stressor(s) represents one of the most robust and reproducible predictors of MDD [21, 22] and the primary paradigm to experimentally induce depressive-like behaviors. Many stress-induced depression models have been developed overtime, the unpredictable chronic mild stress (UCMS) being one of the most commonly used, because of its high face validity (similar phenotype as in depressed patients), construct validity (similar risk factors) and predictive validity (positive response to treatments routinely used in humans) [23]. UCMS-induced depressive-like behaviors, which are usually reversed following chronic treatment with most classical antidepressants, have been primarily linked to hypothalamo–pituitary–adrenal (HPA) axis stimulation and related neurotoxicity [23, 24]. In addition, some studies also report activation of inflammatory processes, but this seems to depend on the stress protocol applied and/or its combination with additional direct immune stimulation [25–27]. Taken together, these findings highlight the high translational potential of the UCMS model and its relevance to study the involvement of different pathophysiological bases of MDD, particularly by modulating stress intensity.

Regarding inflammation-driven depressive-like behaviors, infection models or direct administration of inflammatory cytokine inducers have largely contributed to unravel the mechanisms linking inflammation to depression [11, 28, 29]. These approaches particularly enabled to show the critical role of indoleamine 2,3-dioxygenase (IDO) [30–33], an enzyme which, upon inflammatory activation, degrades tryptophan (TRP) into kynurenine (KYN) at the expense of serotonin (5-HT), a key factor in MDD pathophysiology. Concurrently, KYN pathway activation can also induce depressive symptoms by promoting glutamate-related neurotoxicity. In line with studies documenting the causal chain of events between excessive and/or unbalanced diets, induction of chronic low-grade inflammation and development of MDD [3, 5, 15], high-fat diet (HFD)-induced obesity has also been used as a relevant and reliable translational model of inflammatory depression [34–37]. As for the UCMS model, HFD-induced depressive-like phenotype develops over several weeks, which reflects the progressive alterations of neuronal networks and therefore closely models pathophysiological mechanisms of MDD. Inflammatory processes activation and associated brain function alterations, including those related to the KYN pathway, have also been reported in HFD models [3, 34, 35, 38]. Moreover, they also display dysregulation of another important pathway for inflammation-driven depressive symptoms, the tetrahydrobiopterin (BH4) pathway that ultimately impairs dopamine (DA) neurotransmission [29, 39, 40], as reported in obesity and MDD [41–44]. HFD models therefore recapitulate most of the neurobiological alterations linked to inflammation. Accordingly, they appear as particularly suitable to study the involvement of affected systems in the development of associated depressive symptoms, while offering the opportunity of considering the potential impact of obesity-related metabolic dysregulations [45].

Based on these findings, comparing the UCMS and HFD models appears as a suitable strategy to dissect the specific effects of inflammation on depressive-like symptoms, provided that experimental conditions used to induce depressive-like behaviors only activate inflammatory processes in the HFD model. This study thus aimed to define and validate the adequate experimental design allowing to investigate different depressive-like symptom dimensions and their neurobiological correlates characterizing inflammation-related vs. inflammation-unrelated depression, respectively. Furthermore, in light of clinical findings suggesting that inflammation may interact with other risk factors, especially environmental stress, to induce MDD [2], we also combined HFD and UCMS. This is also relevant in the perspective of studying the impact of this combination on the therapeutic response, since it has been reported to be impaired in these conditions [46], but the underlying neurobiological mechanisms have not yet been investigated. Altogether, this study validated an innovative experimental approach particularly suitable to study inflammation-driven depression and to further assess its likeliness to respond to antidepressant strategies in future investigations.

## Methods

Details are provided in Additional file 1.

### Animals and UCMS procedure

**Fig. 1:**
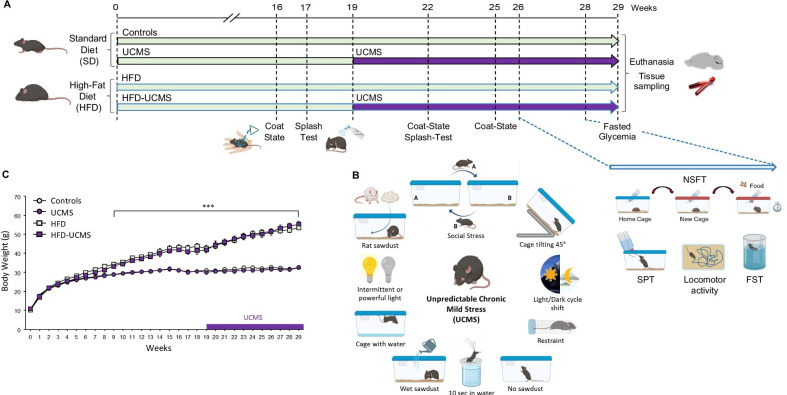
HFD mice displayed expected overweight and related metabolic dysregulations as compared to SD-fed mice. **A** Experimental timeline and design. Male C57BL/6J mice (3 weeks old) were fed ad libitum as soon as they arrived and during the entire experiment with standard diet (SD; 2.9 kcal/g) or high-fat diet (HFD; 5.24 kcal/g). Behavioral measures included: coat-state, splash-test, novelty suppressed feeding test (NSFT), sucrose preference test (SPT), locomotor activity and forced swim test (FST). *n* = 32/group before UCMS onset and 14–16/group after. **B** Schematic representation of the different stressors randomly applied during the UCMS procedure. **C** Body weight was recorded weekly for the entire experiment. It became significantly higher in HFD mice than in their SD counterparts from the 9th week. ^***^P < 0.001 for Diet effect

All procedures were in accordance with European Directives (2010/63/EU) and approved by the Institutional Animal Health and Care Committee (Approval ID: A13169). Upon arrival, 3-week-old male C57BL/6J mice (Janvier Labs, France) were collectively housed under a normal 12 h/12 h light/dark cycle and randomly allocated to standard diet (SD, A04, SAFE, France; 2.9 kcal/g) or HFD groups (D12492, Research Diets, New Brunswick, NJ; 5.24 kcal/g, 60% Kcal from fat), with free access to water and food (Fig. 1A). They were fed with their respective food upon arrival. The UCMS procedure began 19 weeks after the experiment onset and was applied until its end, except the days preceding behavioral tests to avoid potential interferences between acute effects of a particular stressor and impact of chronic stress on mice behavior. Unstressed mice remained group-housed unless transient isolation was required for specific behavioral tests. In this case, they were placed in individual cages 2 days before performing the test, in order to let them habituate to these new housing conditions, and put back to collective cages immediately after. Stressed mice were individually housed during the entire procedure, which consisted to randomly apply several times a day different stressors (e.g., cage tilting, changes of housing conditions or light cycle, social stress, restraint stress; Fig. 1B) following a schedule changed weekly to prevent habituation (see Additional file 2: Table S1).

### Behavioral characterization

Behavioral characterization was performed using well-validated tests allowing to assess different symptom dimensions of MDD: neurovegetative changes (coat-state, splash-test, locomotor activity), depressive-like behaviors (sucrose preference test, SPT; forced swim test, FST) and anxiety-like behaviors (novelty suppressed feeding test, NSFT). Methods were essentially similar to those described previously [34, 47, 48] and are detailed in Additional file 1. Each mouse was submitted to a maximum of 2 behavioral tests per week, with a between-test interval of at least 3 days. To reduce the number of tests per mouse, 2 cohorts that followed the same experimental protocol before behavioral characterization were used. The 1st was tested in the coat-state, splash-test, SPT and FST, while locomotor activity and NSFT were assessed in the 2nd. All biochemical measures were performed in the 1^st^ set of mice, except brain monoamines assays. Behavioral tests were conducted only once, except for the coat-state and splash-test (Fig. 1A).

### Tissue sampling and biochemical measures

Fasted glycemia was measured in 6-h-fasted mice at the end of the UCMS procedure as previously described [49]. One week after completion of behavioral testing, mice were anesthetized by isoflurane inhalation and blood samples immediately collected via cardiac puncture [49]. Commercial kits were used to assay plasma corticosterone (Corticosterone-HS kit; ImmunoDiagnostic System, France), leptin, resistin and adiponectin concentrations (Metabolic- and Adiponectin-Milliplex kits; Merck-Millipore, France). Plasma chemokine and cytokine assays were conducted by Eve Technologies (Calgary, Canada) using a bead-based multiplex assay (Mouse Cytokine/Chemokine Array 32-Plex [MD32]). After blood collection, mice were perfused with chilled 1X PBS and part of the brains rapidly dissected to collect and immediately frozen the hippocampus (HC), prefrontal cortex (PFC) and striatum. After homogenization, DA, 5-HT and their main metabolites (dihydroxyphenyl acetic acid (DOPAC), homovanillic acid (HVA), 5-hydroxyindoleacetic acid (5-HIAA)) were measured by HPLC-EC [50]. Brains used to measure mRNA expression were directly stored at − 80 °C until they were micropunch-dissected as previously described [51].

### Taqman low-density arrays (TLDA)

Total RNAs from HC and PFC micropunches were extracted using Trizol (Invitrogen, Life Technologies, France) and reversed-transcribed to cDNA using the SuperScript-VILO™ cDNA Synthesis Kit (Invitrogen, Thermo-Fisher Scientific, France). A custom-made TLDA card (Applied Biosystems, France) was designed to measure the expression of 48 genes (Additional file 3: Table S2) and processed at the Integrative Microgenomic platform (@BRIDGe, INRA, Jouy-en-Josas, France) following the manufacturer’s protocol. All reactions were performed in duplicates and the relative mRNA expression was normalized against the endogenous controls using the comparative delta–delta Ct method.

### Statistical analysis

Following the method described previously [50, 52], we applied *z*-normalization across data obtained in the coat-state, splash-test, SPT and FST (all performed in the same mice) to calculate an integrated emotionality *z*-score representing a relevant index of the severity of HFD- and UCMS-induced depression-like behaviors. Z-normalization was also applied across complementary measures of plasma, HC and PFC inflammation, KYN and BH4 pathways, 5-HT and glutamate systems, and oxidative status. Regarding inflammatory *z*-scores, both inflammatory and anti-inflammatory factors, which contribute together to the inflammatory response, were integrated in order to better reflect what happens in conditions of chronic inflammation. For the same reasons, the KYN *z*-score included the enzymes promoting either neurotoxicity or neuroprotection. Depending on their distribution, data were analyzed using parametric statistics (two-way ANOVAs with repeated measures for the time factor and post hoc Fisher’s LSD test when appropriate) or non-parametric statistics (Kruskal–Wallis test and Dunn’s pairwise multiple comparison test).

## Results

### HFD mice displayed overweight and related metabolic dysregulations

As expected, HFD mice became progressively heavier than SD mice regardless of stress (Diet: *F*_(1,56)_ = 166.2, *P* < 0.001; Diet × Time: *F*_(5,5280)_ = 51.5, *P* < 0.001; Fig. 1C) and displayed significantly higher plasma leptin (*F*_(1,55)_ = 298.8, *P* < 0.001), resistin (*F*_(1,55)_ = 12.9, *P* < 0.001) and fasted glucose concentrations (*F*_(1,56)_ = 58.8, *P* < 0.001; Additional file 4: Table S3). UCMS increased glycemia (*F*_(1,56)_ = 12.5, *P* < 0.001) and tended to enhance plasma corticosterone levels (*P* = 0.06) whatever the diet.

### HFD and UCMS induced emotional alterations

**Fig. 2:**
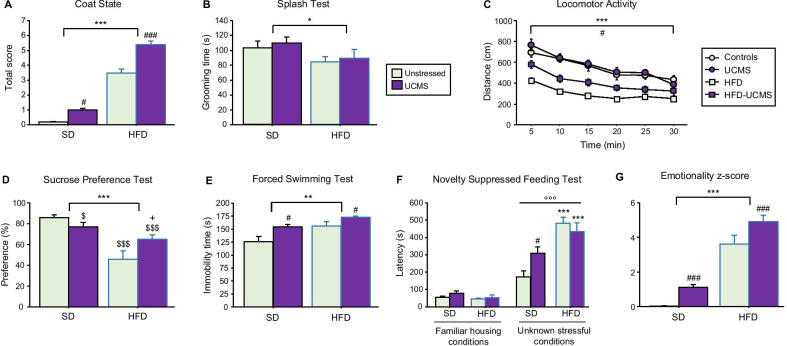
Both HFD and UCMS induced emotional alterations. **A** Total coat-state score assessed week 25 and calculated by summing, for each mouse, the scores given for different body parts that ranged from 0 for a well-groomed coat to 2 for a dirty coat. Each group was significantly different from the others, HFD-UCMS mice displaying the highest score, meaning lower self-care (*n* = 14–16 mice/group). **B** Duration of grooming measured in the splash-test after 4 weeks of UCMS (*n* = 14–16 mice/group). **C** Time-course of the distance traveled in a new cage over the 30-min test. (*n* = 10–11 mice/group). **D** Sucrose preference, whose reduction reflects increased anhedonia, was calculated as the percentage of sucrose intake over the total fluid (sucrose + water) intake. (*n* = 14–16 mice/group). **E** Time spent immobile in the FST (*n* = 14–16 mice/group). **F** Latency to start eating after a 24 h-fasting period when the NSFT was conducted either in the familiar housing conditions (mice home cages and room) or unknown stressful conditions (new cages without bedding placed in an unknown room brightly illuminated). Delayed food intake in these last conditions, as compared to the familiar ones, was used as an index of anxiety (*n* = 10–11 mice/group). **G** Emotionality *z*-score calculated from data obtained in the coat-state, splash-test, SPT and FST (*n* = 14–16 mice/group). Data are graphed as means ± SEM. ^*^P < 0.05, ^**^P < 0.01, ^***^P < 0.001 for Diet effect; ^#^*P* < 0.05, ^###^*P* < 0.001 for Stress effect; ^$^*P* ≤ 0.05, ^$$$^*P* < 0.001 for differences *vs.* unstressed-SD mice; ^+^*P* < 0.05 for differences *vs.* unstressed-HFD mice; ^°°°^*P* < 0.001 for effect of test conditions in the NSFT

Neurovegetative changes were evaluated using the coat-state and splash-test, two paradigms related to self-care and classically used to characterize rodent depression models [48, 53]. Both HFD and UCMS degraded the coat-state, as revealed by the increased scores calculated for those groups (*P* < 0.001; Fig. 2A), these effects being exacerbated when both conditions were combined (*P* < 0.001). In the splash-test, HFD mice groomed less than SD mice (*F*_(1,56)_ = 4.9, *P* < 0.05; Fig. 2B), whereas behavior was unchanged by UCMS. Assessment of locomotor activity was used as an index of psychomotor changes (agitation/retardation) that are classically reported in MDD. It progressively decreased in all mice (Time: *F*_(5,195)_ = 60.0, *P* < 0.001), reflecting the habituation that follows the initial exploration phase due to novelty. In addition, covered distance was reduced by HFD (*F*_(1,39)_ = 65.7, *P* < 0.001; Diet × Time: *F*_(5,195)_ = 3.5, *P* < 0.01; Fig. 2C), but this effect was damped by UCMS (*F*_(1,39)_ = 6.3, *P* < 0.05).

Anhedonia-related depressive-like behaviors were measured in the SPT. HFD significantly decreased sucrose preference (*F*_(1,56)_ = 26.3, *P* < 0.001; Fig. 2D), but to different ranges depending on stress conditions (Diet × Stress: *F*_(1,56)_ = 8.0, *P* < 0.01). UCMS slightly reduced sucrose preference in SD mice, while blunting diet effect in stressed-HFD mice, which still displayed, however lower preference than unstressed controls. In line with these data, increased depressive-like behaviors were also reported in the FST, immobility time being enhanced both by HFD (*F*_(1,49)_ = 7.6, *P* < 0.01; Fig. 2E) or UCMS (*F*_(1,49)_ = 6.3, *P* < 0.05). Of note, this was not just an unspecific consequence of impaired locomotor activity related to overweight since no significant differences were found between groups when swimming was assessed during the first minute of the FST, during which active behavior is classically very high (data not shown). Moreover, there was no significant correlation between body weight and duration of immobility.

The NSFT was used to assess anxiety-like behavior. Latency to eat in a novel environment was increased by HFD (*F*_(1,34)_ = 24.5, *P* < 0.001; Fig. 2F), in interaction with UCMS (*F*_(1,34)_ = 4.5, *P* < 0.05) that specifically enhanced this latency in SD mice. Importantly, this measure was similar in all groups when tested in their home cage (Fig. 2F) and not correlated with food intake assessed in each condition. Together, these results discard potential implication of differences in appetite and therefore confirm increased anxiety-like behavior in HFD and UCMS.

In summary, both experimental conditions induced emotional alterations related to distinct symptom dimensions of MDD. Supporting this, HFD (*F*_(1,56)_ = 146.6, *P* < 0.001) and UCMS (*F*_(1,56)_ = 14.3, *P* < 0.001) independently increased the emotionality *z*-score (Fig. 2G). Of note, this effect appears however to be stronger in HFD than UCMS mice, which suggests that the neurobiological correlates of those behavioral alterations may be also differentially impacted.

### Emotional alterations were associated with activation of inflammatory processes in HFD mice

**Fig. 3:**
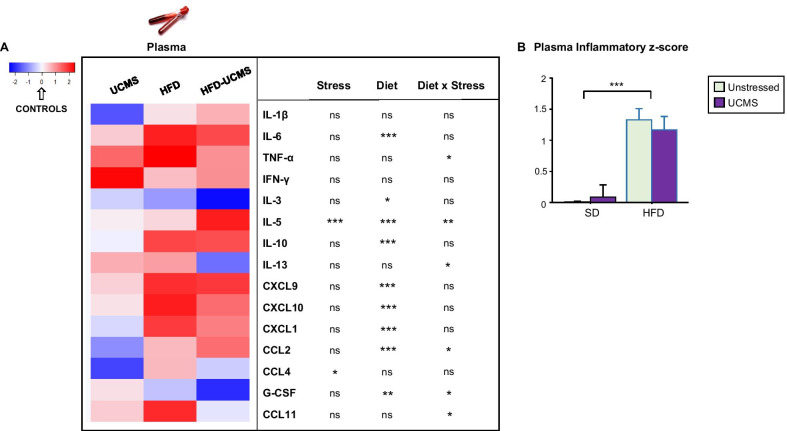
Chronic HFD exposure increased circulating concentrations of inflammatory factors. Plasma levels of cytokines and chemokines analyzed at the end of the experiment in unstressed (Controls) or stressed (UCMS) SD and HFD mice. **A** Heatmap generated with R gplots package showing relative plasma levels (as compared to the control group) of detected inflammatory factors displaying notable differences between groups. Controls are not presented since their value was equal to 0 (white color). The table associated to the heatmap shows results of the statistical analysis reporting the effects of Diet, Stress and their interactions. ^*^P < 0.05 ^**^P < 0.01, ^***^P < 0.001; ns = not significant. **B** Integrated plasma inflammatory *z*-score calculated from data displayed in the heatmap and graphed as means ± SEM. *n* = 8–15 mice/group. ^***^P < 0.001 for Diet effect

To determine whether inflammatory status was differentially affected by HFD and UCMS, plasma levels of a large panel of inflammatory markers were measured. Consistent with the expected obesity-related systemic inflammation, the plasma inflammatory *z*-score calculated from data displayed in Fig. 3A was significantly increased by HFD (*F*_(1,46)_ = 45.1, *P* < 0.001; Fig. 3B) but unchanged by UCMS. HFD particularly enhanced plasma levels of interleukin-6 (IL-6), IL-10, monokine induced by IFN-γ (MIG or CXCL9), IFN-γ-induced protein-10 (IP10 or CXCL10), keratinocytes-derived chemokine (KC or CXCL1) regardless of stress, as well as tumor necrosis factor-α (TNF-α) and eotaxin (CCL11) in unstressed-HFD mice (Fig. 3A and Additional file 5: Fig. S1). When acting, UCMS mainly reduced inflammation, as shown for levels of macrophage inflammatory protein-1β (MIP-1β or CCL4) whatever the diet, monocyte chemoattractant protein-1 (MCP-1 or CCL2) in SD mice, IL-13 and granulocyte-colony stimulating factor (G-CSF) in stressed-HFD mice, although it increased IL-5 levels in the latter.

**Fig. 4:**
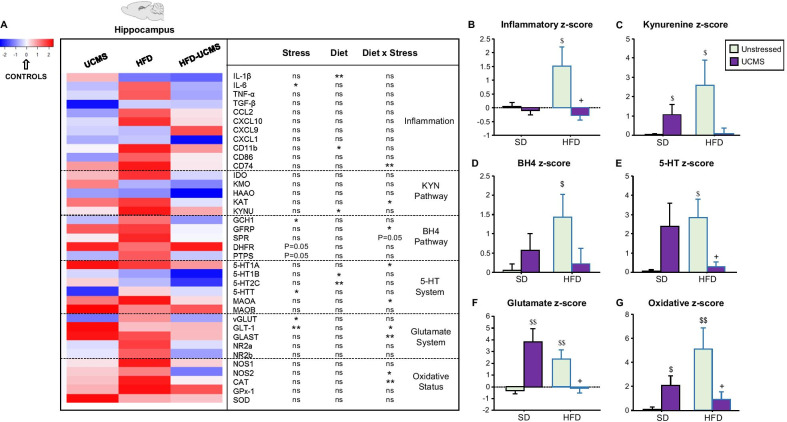
HFD and UCMS differentially modulated HC gene expression of inflammatory markers and related neurobiological processes. **A** Heatmap generated with R gplots package displaying differential expression levels of hippocampus (HC) genes, as analyzed with the TLDA method in unstressed (Controls) or stressed (UCMS) SD and HFD mice. Results are plotted as fold changes relative to controls, which are not presented since their value was equal to 0 (white color). Genes showing poor or late amplification were not included in the analysis. The table associated to the heatmap displays results of the statistical analysis for genes related to: inflammation, KYN pathway, BH4 pathway, 5-HT system, glutamate system and oxidative status. ^*^P < 0.05 ^**^P < 0.01; ns = not significant. **B** Inflammatory, **C** kynurenine, **D** BH4, **E** 5-HT, **F** glutamate and **G** oxidative *z*-scores calculated from expression of related genes displayed in the heatmap. All results are graphed as means ± SEM. *n* = 8–10 mice/group. ^$^*P* ≤ 0.05, ^$$^*P* < 0.01 for differences vs. unstressed-SD mice; ^+^*P* ≤ 0.05 for differences vs. unstressed-HFD mice

**Fig. 5:**
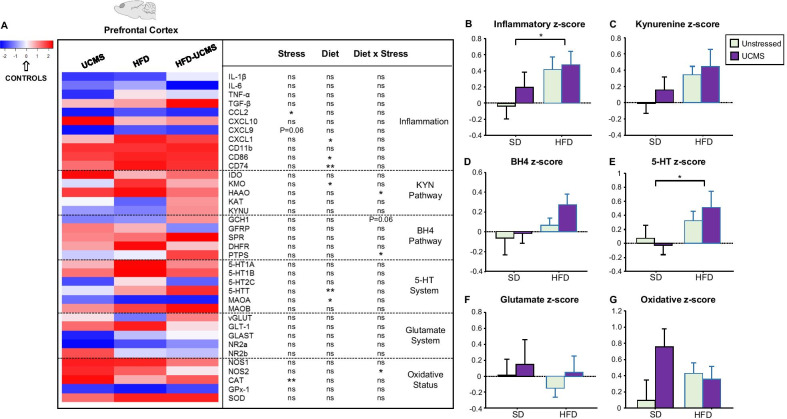
HFD and UCMS differentially modulated PFC gene expression of inflammatory markers and related neurobiological processes. (**A**) Heatmap generated with R gplots package displaying differential expression levels of prefrontal cortex (PFC) genes, as analyzed with the TLDA method in unstressed (Controls) or stressed (UCMS) SD and HFD mice. Results are plotted as fold changes relative to controls, which are not presented since their value was equal to 0 (white color). Genes showing poor or late amplification were not included in the analysis. The table associated to the heatmap displays results of the statistical analysis for genes related to: inflammation, KYN pathway, BH4 pathway, 5-HT System, glutamate system and oxidative status. ^*^P < 0.05 ^**^P < 0.01; ns = not significant. (**B**) Inflammatory, (**C**) kynurenine, (**D**) BH4, (**E**) 5-HT, (**F**) glutamate and (**G**) oxidative *z*-scores calculated from expression of related genes displayed in the heatmap. (*n* = 8–10 mice/group). All results are graphed as means ± SEM. ^*^P < 0.05 for Diet effect

Akin to peripheral inflammation, HFD upregulated gene expression of several markers of microglial activation in two central sites for emotional regulation, the HC (Fig. 4A and Additional file 6: Fig. S2A) and PFC (Fig. 5A and Additional file 7: Fig. S3A) [49, 54]. Accordingly, the HC inflammatory *z*-score (integrating data from inflammatory factors displayed in Fig. 4A) was significantly increased in unstressed-HFD mice (Diet × Stress: *F*_(1,36)_ = 5.1, *P* < 0.05; Fig. 4B), UCMS counteracting the effect of HFD (*F*_(1,36)_ = 7.1, *P* < 0.05). In the PFC, HFD significantly enhanced local inflammatory *z*-score (*F*_(1,36)_ = 4.4, *P* < 0.05; Fig. 5B), independently from stress. Altogether, these data confirmed activation of inflammatory processes in HFD mice, but not unstressed- or stressed-SD mice, while highlighting stress-related anti-inflammatory properties in the HC of HFD-UCMS mice.

### HFD-induced inflammation was associated with modulation of KYN and BH4 pathways

We then assessed the impact of HFD and UCMS on the KYN and BH4 pathways (Figs. 4A, 5A), which contribute to inflammation-related depression [29, 40]. In the HC, the KYN *z*-score, which integrated the expression of related enzymes (IDO, kynurenine aminotransferase (KAT), kynurenine 3-monoxygenase (KMO), kynureninase (KYNU) and hydroxyanthranilic acid oxygenase (HAAO)), was increased in stressed-SD and unstressed-HFD mice (Diet × Stress: *F*(_1,36)_ = 6.5, *P* < 0.05; Fig. 4C). Interestingly, while UCMS particularly targeted KAT expression in SD mice (Diet × Stress: *F*_(1,28)_ = 4.6, *P* < 0.05; Additional file 6: Fig. S2B), HFD rather promoted KYN-related neurotoxicity by enhancing KYNU expression (*F*_(1,31)_ = 4.2, *P* < 0.05). BH4 *z*-score integrating main BH4-related enzymes and regulatory proteins (guanosine triphosphate cyclohydrolase-1 (GCH1), GTP-cyclohydrolase-1 feedback regulator (GFRP), sepiapterin reductase (SPR), dihydrofolate reductase (DHFR) and 6-pyruvoyltetrahydropterin synthase (PTPS)) was also notably increased in unstressed-HFD mice (Stress × Diet: *F*_(1,36)_ = 4.4, *P* < 0.05; Fig. 4D). UCMS and HFD indeed interacted to differentially modulate GFRP (*F*_(1,31)_ = 5.2, *P* < 0.05; Additional file 6: Fig. S2C) and SPR expression (*F*_(1,35)_ = 4.0, *P* = 0.05). UCMS decreased expression of GCH1 (*F*_(1,33)_ = 4.6, *P* < 0.05) and PTPS (*F*_(1,33)_ = 3.9, *P* = 0.05), while increasing that of DHFR (*F*_(1,34)_ = 4.1, *P* = 0.05). Consistent with KYN *z*-score, both factors modulated glutamatergic neurotransmission in stressed-SD and unstressed-HFD mice, as revealed by enhancement of glutamate *z*-score (Diet × Stress: *F*_(1,36)_ = 19.0, *P* < 0.001; Fig. 4F) that integrated expression of glutamate transporters (vesicular glutamate transporter (vGLUT), glutamate transporter-1 (GLT-1), glial high-affinity glutamate transporter (GLAST)) and NMDA receptor subunits (NR2a, NR2b). Of note, vGLUT expression was significantly down-regulated by UCMS (*F*_(1,32)_ = 5.2, *P* < 0.05; Additional file 6: Fig. S2E), which interacted with HFD to up-regulate that of GLT-1 (Stress: *F*_(1,31)_ = 7.9, *P* < 0.01; Diet × Stress: *F*_(1,31)_ = 7.2, *P* < 0.05) and GLAST (Diet × Stress: *F*_(1,35)_ = 7.8, *P* < 0.01). Lastly, the oxidative *z*-score calculated from catalase (CAT), superoxide dismutase-1 (SOD), glutathione peroxidase-1 (GPx-1), nitric oxide synthase-1 (NOS1) and NOS2 expression was also increased in the same two groups (Diet: *F*_(1,36)_ = 3.7, *P* = 0.06; Diet × Stress: *F*_(1,36)_ = 12.6, *P* < 0.01; Fig. 4G), CAT and NOS2 expression being significantly upregulated by HFD, while UCMS blunted this effect (Diet × Stress: *F*_(1,33)_ = 8.4, *P* < 0.01 and *F*_(1,34)_ = 5.4, *P* < 0.05, respectively; Additional file 6: Fig. 2F).

In the PFC, the impact of HFD on KYN *z*-score did not reach significance (*F*_(1,36)_ = 3.6, *P* = 0.07; Fig. 5C), but it increased KMO (*F*_(1,35)_ = 9.9, *P* = 0.05; Additional file 7: Fig. S3B) and HAAO expression (Diet × Stress: *F*_(1,36)_ = 5.6, *P* < 0.05), which was also enhanced in stressed-SD mice. Supporting further HFD-induced KYN-related neurotoxicity, the neurotoxicity/neuroprotection ratio (KMO/KAT) was significantly increased by HFD (*F*_(1,33)_ = 5.0, *P* < 0.05; Additional file 7: Fig. S3B) and reduced by UCMS (*F*_(1,33)_ = 7.1, *P* < 0.05). BH4 *z*-score was similar in all mice (Fig. 5D), although UCMS and HFD interacted to increase PTPS expression in stressed-HFD mice (*F*_(1,35)_ = 5.2, *P* < 0.05; Additional file 7: Fig. S3C) that also tended to display overexpression of GCH1 (*F*_(1,36)_ = 3.6, *P* = 0.06). Lastly, glutamate and oxidative *z*-scores were not significantly changed (Fig. 5F, G), but UCMS increased CAT expression (*F*_(1,34)_ = 8.4, *P* < 0.01; Additional file 7: Fig. S3E) and interacted with HFD to upregulated that of NOS2 (*F*_(1,35)_ = 4.5, *P* < 0.05).

### Changes in monoaminergic neurotransmission accompanied modulation of KYN and BH4 pathways

Because 5-HT system participates to MDD pathophysiology and can be impacted by inflammation and related modulation of KYN and BH4 pathways, we measured whether UCMS and HFD affected gene expression of key 5-HT elements (5-HT1A, 1B, 2C receptors, 5-HT transporter (5-HTT) and monoamine oxidases (MAO) degradation enzymes; Figs. 4A, 5A). In the HC, the 5-HT *z*-score calculated from these elements was increased in stressed-SD and unstressed-HFD mice (Stress × Diet: *F*_(1,36)_ = 8.9, *P* < 0.01; Fig. 4E). Accordingly, these mice displayed increased 5-HT1A and MAOA expression (Stress × Diet: *F*_(1,31)_ = 4.2, *P* < 0.05 and *F*_(1,30)_ = 6.3, *P* < 0.05, respectively; Additional file 6: Fig. S2D) and reduced 5-HT concentrations (Stress × Diet: *F*_(1,31)_ = 4.2, *P* < 0.05; Table 1), while 5-HIAA levels and 5-HIAA/5-HT ratio were unchanged. Conversely, HFD decreased 5-HT1B (*F*_(1,36)_ = 4.8, *P* < 0.05; Additional file 6: Fig. S2D) and 5-HT2C expression (*F*_(1,31)_ = 8.9, *P* < 0.01) and UCMS that of 5-HTT (*F*_(1,31)_ = 6.4, *P* < 0.05). In the PFC, HFD increased 5-HT *z*-score (*F*_(1,36)_ = 4.9, *P* < 0.05; Fig. 5E) and 5-HTT expression (*F*_(1,30)_ = 7.8, *P* < 0.01; Additional file 7: Fig. S3D) regardless of stress, while slightly reducing MAOA expression (*F*_(1,32)_ = 4.4, *P* < 0.05). No significant changes of 5-HT and 5-HIAA levels or their ratio were, however, reported (Table 1).

**Table 1:** Impact of HFD and UCMS on brain concentrations of monoamines and their metabolites

(pmoles/g tissue)		Controls	UCMS	HFD	HFD-UCMS
Hippocampus	[5-HT]	2031.5 ± 189.9	1688.0 ± 163.3	1487.1 ± 141.6*	1934.3 ± 280.2
	[5-HIAA]	2044.0 ± 146.6	2084.0 ± 124.4	2610.4 ± 477.8	2297.3 ± 177.4
	5-HIAA/5-HT	1.1 ± 0.2	1.3 ± 0.2	1.6 ± 0.3	1.4 ± 0.2
	[DA]	57.7 ± 6.9	69.0 ± 14.0	116.5 ± 34.3*	171.2 ± 51.5*
	[DOPAC]	87.6 ± 11.7	77.5 ± 6.9	95.7 ± 13.6	120.2 ± 29.9
	[HVA]	ND	ND	ND	ND
	DOPAC/DA	1.5 ± 0.2	1.7 ± 0.4	1.1 ± 0.2	1.0 ± 0.2
Cortex	[5-HT]	1662.7 ± 83.4	1657.6 ± 80.7	1505.8 ± 75.8	1717.2 ± 95.9
	[5-HIAA]	1939.8 ± 178.4	1741.1 ± 169.7	1559.5 ± 199.2	1794.2 ± 141.3
	5-HIAA/5-HT	1.2 ± 0.1	1.1 ± 0.1	1.0 ± 0.1	1.1 ± 0.1
	[DA]	6828.2 ± 1140.7	8175.8 ± 1676.7	9125.4 ± 673.7	7866.6 ± 1164.1
	[DOPAC]	2164.2 ± 181.6	2021.4 ± 296.9	2545.4 ± 131.4	2394.0 ± 277.8
	[HVA]	1450.0 ± 183.8	1486.8 ± 128.0	1666.5 ± 115.8	1686.2 ± 160.9
	DOPAC/DA	0.4 ± 0.09	0.5 ± 0.2	0.3 ± 0.03	0.4 ± 0.05
	HVA/DA	0.3 ± 0.1	0.2 ± 0.05	0.2 ± 0.02	0.2 ± 0.04
Striatum	[5-HT]	1216.3 ± 220.7	1360.6 ± 172.0	1473.2 ± 219.4	1267.2 ± 223.0
	[5-HIAA]	2616.5 ± 472.5	1972.9 ± 258.6	2850.3 ± 269.4*	2484.1 ± 276.4*
	5-HIAA/5-HT	2.2 ± 0.3	1.7 ± 0.2	2.2 ± 0.3	2.6 ± 0.5
	[DA]	19,358.3 ± 4421.4	10,115.5 ± 2406.4^#^	18,595.6 ± 3641.0	13,783.9 ± 3034.9^#^
	[DOPAC]	3639.0 ± 541.3	3331.3 ± 633.5	3806.8 ± 393.6	3365.9 ± 629.9
	[HVA]	2374.3 ± 500.1	2443.6 ± 366.1	2965.4 ± 291.9	2789.7 ± 307.4
	DOPAC/DA	0.3 ± 0.05	0.5 ± 0.1	0.3 ± 0.08	0.3 ± 0.09
	HVA/DA	0.1 ± 0.02	0.3 ± 0.1	0.2 ± 0.1	0.3 ± 0.04

Concentrations of 5-HT, DA and their metabolites (DOPAC and HVA for DA; 5-HIAA for 5-HT) measured by HPLC-EC at the end of the experiment in the HC, PFC and Striatum of unstressed (Controls) or stressed (UCMS) SD and HFD mice. Values are expressed as pmoles/g of tissue. *n* = 10–11 mice/group. ND: not detectable. ^*^P < 0.05 for diet effect; ^#^*P* < 0.05 for stress effect

Because impaired DA neurotransmission has been reported in obesity and MDD [41–44], DA and its metabolites were measured in the HC, PFC, but also the striatum as an important dopaminergic site (Table 1). No significant changes were reported for DA levels in the PFC, but they were decreased by UCMS in the striatum (*F*_(1,36)_ = 4.6, *P* < 0.05) and increased by HFD in the HC (*F*_(1,34)_ = 6.5, *P* < 0.05), although they remained much lower here than in the other regions. Lastly, DA metabolite levels were similar whatever the groups.

**Fig. 6:**
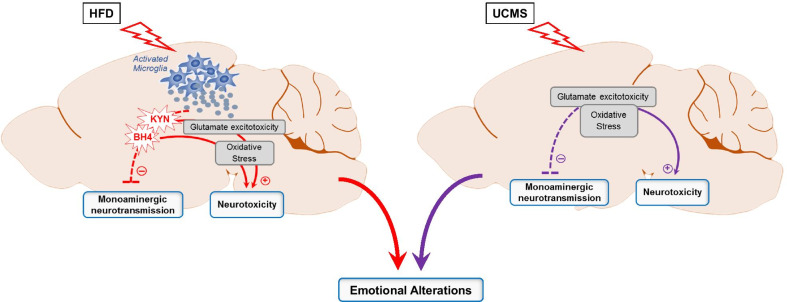
Overview of the neurobiological processes, associated with HFD-induced and UCMS-induced emotional alterations respectively. In line with the initial expectations, inflammation was selectively reported in the model of HFD-related depression, as shown by increased expression of markers of microglial activation in the HC and PFC. Consistent with this, HFD mice also displayed activation of the KYN and BH4 pathways, which are known to trigger inflammation-driven depression. This activation, which was particularly sustained in the HC, was associated with impaired local monoaminergic neurotransmission, notably the 5-HT system. It concomitantly promoted glutamate excitotoxicity and oxidative stress, favoring in turn neurotoxicity. Of note, these neurobiological changes that likely contribute to emotional alterations were also found in the UCMS depression model, but importantly, upstream triggering mechanisms were in that case independent from inflammation. The two experimental conditions enable, respectively, to dissociate inflammation-related vs. inflammation-unrelated depressive symptoms. Arrows indicate activation and dotted T-bars inhibition/impairment

Taken together, these results showed that emotional alterations instigated by HFD and UCMS were associated with differential induction of systemic and brain inflammation, specifically reported in HFD mice, distinct activation of KYN and BH4 pathways, particularly in the HC, together with increased oxidative status and dysregulated brain glutamate and monoaminergic neurotransmission (Fig. 6). In addition, they pointed to a particular regulation of HFD-induced inflammatory activation and related neurobiological alterations in the HC of HFD-UCMS mice.

## Discussion

Dissecting the relative contribution of inflammatory processes in the occurrence of MDD remains a challenge in the field of immunopsychiatry, and the lack of suitable preclinical models of depression further complicates this issue. This study provides valuable findings relevant to this topic by validating an experimental strategy that enables dissociating inflammation-related *vs.* inflammation-unrelated depressive-like behaviors and to decipher the respective cascade of events underlying their induction.

While most depression models usually focus on one particular MDD risk factor [23, 55, 56], we compared exposure to HFD and UCMS. Both paradigms induced neurovegetative alterations resembling MDD symptoms, including apathy and carelessness [36, 48, 57, 58], as notably evidenced by increased coat-state score. As HFD is greasy and friable, this could explain fur degradation in HFD mice. However, it is noteworthy that similar observation was reported in stressed-SD mice. Importantly, the splash-test confirmed impaired grooming in HFD mice, as previously shown in obese mice and other depression models [37, 58–61]. Regarding UCMS, published data changed depending on mouse strains used, and the intensity, nature and/or duration of the stress protocol [23, 46, 48, 53, 61]. Here, C57BL/6J mice were chosen as classical strain for HFD-induced obesity studies, although they are not the most responsive to UCMS [34, 37, 42, 44]. Moreover, strong stressors such as food and water deprivation were discarded for ethical reasons. This likely explains why stressed-SD mice behaved as controls in the splash-test, while otherwise displaying higher emotional behaviors. As previously shown [44, 50, 61, 62], UCMS and HFD notably increased immobility in the FST. Although it could be postulated that HFD-related locomotor impairment may be a confounding factor in this test [45], changes in immobility likely reflected depressive-like behavior, consistent with impaired sucrose preference in the SPT, a reward-based test modeling anhedonia, which does not rely on locomotor response. Further ruling out potential interferences of motor impairment in the FST, we previously showed that changes in immobility can be reported in this test without general locomotion necessarily being altered [30–33]. In line with this, HFD-induced anxiety-like behavior, as assessed in behavioral tests involving locomotor response, were also independent of overall locomotion [45]. Akin to these findings, we showed here that both HFD and UCMS mice displayed prolonged latency to eat in the NSFT only when conducted in the new environment, which reflects increased anxiety-like behaviors unrelated to changes in appetite or locomotion [37, 47, 48, 50, 62]. Altogether, these data therefore confirmed that HFD and UCMS models displayed depressive phenotypes, although they did not necessarily induce similar behavioral alterations. Of note, a specific behavioral profile was also reported when the two conditions were combined, at least regarding some depressive dimensions. This agrees with previously published data [46], which interestingly show that this was associated with a differential response to chronic antidepressant treatment, supporting further the interest of having several complementary preclinical models to study the pathophysiological bases of TRD.

As anticipated, both depression models displayed different neurobiological changes potentially contributing to their behavioral phenotype. This could include metabolic dysregulations specifically induced by HFD, as previously reported [45, 63], but mounting evidence suggests that they unlikely play a direct predominant role. Indeed, impaired emotional behaviors were previously associated with low leptin levels or increased leptin resistance [38, 64, 65], while mice with high leptin levels, but no inflammation, exhibit normal depressive-like behavior [34]. Moreover, improving obesity-driven inflammation and emotional alterations can be achieved without concomitantly normalizing adipokine and/or glucose levels, and *vice-versa* [49, 53, 66, 67], although some studies report positive behavioral effects of anti-diabetic drugs [37, 68]. Of note however, these drugs may act by reducing, beyond hyperglycemia, inflammation [69, 70]. Here, elevated plasma levels of inflammatory factors contributing to the overall innate immune system activation [15, 34, 38, 42, 64, 71] and increased brain expression of classical markers of microglial activation [36, 64, 72] were selectively triggered by HFD. These results were in line with a wide literature also reporting other compelling signs of inflammation and immune activation related to obesity, such as adipose tissue production of inflammatory factors or local infiltration of activated immune cells [71, 73, 74]. Unlike other studies using stress protocols stronger than ours, different strains of mice and/or additional immune stimulations [25, 26, 75], stressed-SD mice were not inflamed, which could likely account for the behavioral differences reported between these mice and unstressed-HFD mice. UCMS even occasionally altered HFD-induced inflammation, as also reported for some emotional behaviors. These results are consistent with the wide, although often conflicting literature illustrating the complexity of the bidirectional relationship between chronic stress and consumption of palatable food [76–80]. Indeed, it has been shown that chronic stress exposure can mitigate the adverse effects of HFD, in particular through the anti-inflammatory properties of stress-induced HPA axis activation [76, 80]. Conversely, HFD consumption has been reported to alleviate the deleterious effects of stress on depressive symptoms and related neurobiological impairments [77–79]. Additional studies are needed to understand further how stress interacts with obesity-driven inflammation and related symptoms in the current experimental conditions. Meanwhile, we clearly showed that UCMS alone did not activate inflammation in our experimental conditions, thus meeting the specifications that were initially set.

Interestingly, both UCMS and HFD models also differed regarding some of the main downstream neurobiological events triggering inflammation-driven depression [3, 5, 11, 29, 40]. This primarily included KYN pathway activation in HFD mice, together with reduced hippocampal 5-HT levels and imbalanced brain neurotoxic–neuroprotective ratio favoring neurotoxicity and oxidative stress, as previously reported in the adipose tissue and plasma of obese patients [81, 82]. Enzymes of the BH4 pathway were also differentially expressed, particularly in the HC of unstressed-HFD mice, with potential impact on monoaminergic neurotransmission, as BH4 is required for optimal DA and 5-HT synthesis [29, 40]. Here, both HFD and UCMS differentially altered those systems in a monoamine-dependent and region-dependent manner, what was expected given their central role in the pathophysiology and treatment of MDD [3, 41]. The mechanisms, respectively, underlying stress-induced and HFD-induced modulation of monoamine neurotransmission and related neuropsychiatric symptoms still need to be deeply studied, what supports further the relevance of comparing the two models.

The current work bears some limitations that upcoming experiments should overcome. The main one is that data obtained are essentially correlative and do not therefore allow concluding about the causal role of the different neurobiological processes studied, as well as their potential links, in the development of reported emotional alterations, nor identifying other possible underlying mechanism, particularly regarding the combined impact of stress and HFD. Of note however, this was not the aim of the present study. Another limitation is that experiments were only performed on males, while women are at greater risk to suffer from MDD [83]. This is due to largely multifactorial reasons [83] that are not necessarily easy to control experimentally and might in any case complicate data interpretation when both sexes are studied together. Being aware of that, and in order to reduce the number of mice used, we decided not to address this issue here. This means that extrapolating the present findings to females needs to be taken with caution. Despite these limitations, the in-depth characterization of the two models of depression used in this study represents an essential first step in the further development of new pharmacological and/or more mechanistic studies.

## Conclusions

In conclusion, by validating an experimental approach allowing the comparative analysis of inflammatory versus non-inflammatory depression models, this study highlights the relevance of this approach to unravel the role of inflammatory processes in the pathophysiology of MDD. It should notably help to identify the nature of inflammation-driven brain alterations specifically involved in the development of particular symptom dimensions and better understand the pathophysiological bases of the clinical phenotype resulting from the combination of stress and HFD. In addition, it should also enable addressing questions on the implication of inflammation in the treatment of those disorders, by comparing the response to antidepressants between the two conditions. In that context, the detailed insights into the behavioral and neurobiological changes they, respectively, induced would be useful for the potential development of new therapeutic strategies, particularly those targeting inflammation. They could also facilitate the identification of reliable phenotypic markers to characterize the profile of patients with TRD. Lastly, unlike studies combining stress and direct immune activation instead of obesity-driven inflammation [25–27], our experimental strategy takes into account an important player in MDD pathophysiology, namely nutritional imbalance and its impact on brain function. Altogether, this study opens new avenues for future research.

## Supplementary Information

**Additional file 1. **Supplementary methods providing detailed descriptions of the UCMS protocol, behavioral testing procedures, multiplex and TLDA assays and Z-scores calculation.

**Additional file 2: Table S1.** Detailed daily schedule of the UCMS protocol.

**Additional file 3: Table S2.** List of genes analyzed in the Taqman low-density arrays (TLDA).

**Additional file 4: Table S3.** Plasma levels of adipokines, corticosterone and glucose measured at the end of the experiment.

**Additional file 5: Fig. S1.** HFD mice displayed expected increase of circulating concentrations of inflammatory factors. Plasma levels of cytokines and chemokines analyzed at the end of the experiment in unstressed (Controls) or stressed (UCMS) SD and HFD mice. Detailed statistical analysis reported significant differences between groups for circulating levels of: (A) IL-6, (B) TNF-α, (C) IL-3, (D) IL-5, (E) IL-10, (F) IL-13, (G) MIG or CXCL9, (H) IP10 or CXCL10, (I) KC or CXCL1, (J) MCP-1 or CCL2, (K) MIP-1β or CCL4, (L) G-CSF and (M) CCL11. *n* = 8–15 mice/group. All results are graphed as means ± SEM. ^**^P < 0.01, ^***^P < 0.001 for Diet effect; ^#^*P* < 0.05 for Stress effect; ^$^*P* < 0.05, ^$$^*P* < 0.01, ^$$$^*P* < 0.001 for differences *vs.* unstressed-SD mice; ^++^*P* < 0.01, ^+++^*P* < 0.001 for differences *vs.* unstressed-HFD mice.

**Additional file 6: Fig. S2.** HFD and UCMS differentially modulated HC gene expression of inflammatory markers and related neurobiological processes. Relative gene expression (as compared to controls) measured by TLDA analysis in the hippocampus (HC) of unstressed (Controls) or stressed (UCMS) SD and HFD mice. Detailed analysis revealed significant impact of HFD and/or UCMS for: (A) inflammatory cytokines and markers of microglial activation (*IL-1β, IL-6, CD11b, CD74*); (B) enzymes from the kynurenine (KYN) pathway (*KAT, KYNU*); (C) enzymes from the tetrahydrobiopterin (BH4) pathway (*GCH1, GFRP, SPR, DHFR, PTPS*); (D) key elements of the 5-HT system (*5-HT1A, 5-HT1B, 5-HT2C* receptors, 5-HT transporter (*5-HTT*), monoamine oxidase A (*MAOA*)); (E) markers of glutamate system (*vGLUT, GLT-1, GLAST*) and (F) oxidative enzymes (*NOS2, CAT*). (*n* = 8–10 mice/group). All results are graphed as means ± SEM. ^*^P < 0.05, ^**^P < 0.01 for Diet effect; ^#^*P* < 0.05, ^##^*P* < 0.01 for Stress effect; ^$^*P* < 0.05, ^$$^*P* < 0.01 for differences *vs.* unstressed-SD mice; ^+^*P* ≤ 0.05 for differences *vs.* unstressed-HFD mice.

**Additional file 7: Fig. S3.** HFD and UCMS differentially modulated PFC gene expression of inflammatory markers and related neurobiological processes. Relative gene expression (as compared to controls) measured by TLDA analysis in the prefrontal cortex (PFC) of unstressed (Controls) or stressed (UCMS) SD and HFD mice. Detailed analysis revealed significant impact of HFD and/or UCMS for: (A) Markers of microglial activation (*CCL2, CXCL9, CXCL1, CD86, CD74*); (B) enzymes from the KYN pathway (*KMO, HAAO, KAT*) and the neurotoxicity/neuroprotection ratio (expression level of *KMO/KAT*); (C) enzymes from the BH4 pathway (*GCH1, PTPS*); (D) key elements of the 5-HT system (*5-HTT, MAOA*) and (E) oxidative enzymes (*NOS2, CAT*). (*n* = 8–10 mice/group). All results are graphed as means ± SEM. ^*^P < 0.05, ^**^P < 0.01 for Diet effect; ^#^*P* < 0.05 for Stress effect; ^$^*P* < 0.05 for differences *vs.* unstressed-SD mice; ^+^*P* < 0.05 for differences *vs.* unstressed-SD mice.

## Data Availability

The datasets used and/or analyzed during the current study are available from the corresponding authors on reasonable request.

## Abbreviations

MDD: Major depressive disorder
TRD: Treatment-resistant depression
UCMS: Unpredictable chronic mild stress
HPA axis: Hypothalamo–pituitary–adrenal axis
IDO: Indoleamine 2,3-dioxygenase
TRP: Tryptophan
KYN: Kynurenine
5-HT: Serotonin
HFD: High-fat diet
BH4: Tetrahydrobiopterin
DA: Dopamine
SD: Standard diet
SPT: Sucrose preference test
FST: Forced swim test
NSFT: Novelty suppressed feeding test
HC: Hippocampus
PFC: Prefrontal cortex
DOPAC: Dihydroxyphenyl acetic acid
HVA: Homovanillic acid
5-HIAA: 5-Hydroxyindoleacetic acid
TLDA: Taqman low-density arrays
IL-6: Interleukin-6
CXCL9: Monokine induced by IFN-γ
CXCL10: IFN-γ-induced protein-10
TNF-α: Tumor necrosis factor-α
CXCL1: Keratinocytes-derived chemokine
CCL4: Macrophage inflammatory protein-1β
CCL11: Eotaxin
CCL2: Monocyte chemoattractant protein-1
G-CSF: Granulocyte-colony stimulating factor
KAT: Kynurenine aminotransferase
KMO: Kynurenine 3-monoxygenase
KYNU: Kynureninase
HAAO: Hydroxyanthranilic acid oxygenase
GCH1: Guanosine triphosphate cyclohydrolase-1
GFRP: GTP-cyclohydrolase-1 feedback regulator
SPR: Sepiapterin reductase
DHFR: Dihydrofolate reductase
PTPS: 6-Pyruvoyltetrahydropterin synthase
vGLUT: Vesicular glutamate transporter
GLT-1: Glutamate transporter-1
GLAST: Glial high-affinity glutamate transporter
CAT: Catalase
SOD: Superoxide dismutase-1
GPx-1: Glutathione peroxidase-1
NOS: Nitric oxide synthase
5-HTT: 5-HT transporter
MAO: Monoamine oxidase
CD11b: Clusters of differentiation 11b
