# Supplementary material for: A new experimental design to study inflammation-related versus non-inflammation-related depression in mice
Source: J Neuroinflammation. 2021 Dec 11;18:290. doi: 10.1186/s12974-021-02330-9 (PMC8666053; doi:10.1186/s12974-021-02330-9)
Supplement: Supplementary file 1 — Additional file 1. Supplementary methods providing detailed descriptions of the UCMS protocol, behavioral testing procedures, multiplex and TLDA assays and Z-scores calculation. [file 12974_2021_2330_MOESM1_ESM.pdf]

A new experimental design to study inflammation-related versus non-inflammation-related depression in mice

Supplementary Methods

Unpredictable chronic mild stress (UCMS). The procedure adapted from Couroussé *et al.* (2015) [1] was daily applied from the 19th week and until the end of the experiment (week 29). From the 26th week of UCMS, the procedure was punctually suspended the day before the behavioral tests to avoid potential interferences between acute effects of a particular stressor and chronic stress impact on mice behavior. Stressed mice were randomly subjected several times a day to one of the following stressors: damp sawdust, repeated sawdust changing, placement in an empty cage or a cage filled with 1cm of water, switching cages between 2 mice (social stress), cage tilting (45°), housing on a bedding from rats or CD1 aggressive mice, exposure to a powerful light (400 lx), inversion of light/dark cycle, intermittent lights on during the dark phase or light off during the light phase, confinement in small tubes (restraint). Of note, the stress procedure did not involve food and water deprivation. In order to prevent habituation, the stressor schedule was changed from week to week, as detailed in Table S1 (see Additional file 2).

Behavioral testing. All behavioral tests were performed in the morning, under conditions of dim light and low noise. Mice were tested in a random order that was changed between each test. Testing equipment was thoroughly cleaned between each session with ethanol solution (30%). For non-automatized tests, scoring was done by trained observers blind to experimental conditions.

- *Coat-state.* This scoring, adapted from Surget et al. (2008) [2], was assessed in different body parts (head, neck, dorsal and ventral coat, tail, forepaws, hindpaws and genital area). The score given for each area ranged from 0 for a well-groomed coat to 2 for a dirty coat, 1 indicating an intermediate state. Total score for each mouse was obtained by summing individual scores and therefore, when high, reflected low self-care.

- *Splash-test.* This test was performed in mice home cages, essentially as described previously (Surget et al. 2008) [2]. A viscous 10% sucrose solution that normally triggers grooming behavior was squirted on the dorsal coat of each mouse. Latency to initiate grooming, as well as its duration, were manually scored over the 5-min test as an index of self-care.

- *Locomotor activity.* To assess locomotor activity, mice were placed in individual cages (30×12cm) filled with fresh bedding under dim light (70lx) and videotracked for 30min using Smart software (Bioseb, France). Total distance travelled was automatically quantified.

- *Sucrose preference test (SPT).* Individually-housed mice were first presented two bottles of tap water to measure basal water consumption and potential place preference. After 72h of habituation (bottle sides reversed daily), one of the bottles was filled with a 2% sucrose solution. Sucrose and water intakes were measured 24h later by weighing the two bottles. Sucrose preference, whose reduction reflects increased anhedonia, was calculated as the percentage of sucrose intake over the total fluid (sucrose + water) intake.

- *Forced swim test (FST).* As previously described [3], mice were placed individually in a cylinder (diameter: 16cm; height: 31cm) half-filled with 25°C+/-1°C water for 6min. Behavior (swimming, climbing and immobility) was videotaped to be scored later by a trained observer using the ‘‘Observer Basic’’ software (Noldus, Netherlands). Increased immobility time was used as an index of despair and helplessness.

- *Novelty suppressed feeding test (NSFT).* The method used was adapted from Dulawa and Hen (2005) [4]. After 24h of fasting in their home cages, mice were placed in novel individual cages of the same size as theirs, but without bedding, and moved to an unknown room brightly illuminated (anxiogenic setup). Pre-weighed food was then provided and the latency to start eating was measured, as well as total food intake at the end of the 2h of test. To control for potential biases due to differences in appetite, mice were re-tested in their home cage after another 24h-fasting period. Delayed food intake in the novel conditions (anxiogenic setup), as compared to home cage, was used as an anxiety index.

Mouse Cytokine/Chemokine Array 32-Plex. Plasma chemokine and cytokine assay was conducted by Eve Technologies (Calgary, Canada) using a bead-based multiplex assay (Mouse Cytokine/Chemokine Array 32-Plex [MD32]). All samples were assayed in simplicate and prepared standards were included in all runs. The following proteins were assessed in the plasma: Interleukin-1α (IL-1α), IL-1β, IL-2, IL-3, IL-4, IL-5, IL-6, IL-7, IL-9, IL-10, IL-12 p40, IL-12 p70, IL-13, IL-15, IL-17, Interferon gamma (IFN- γ), Tumor necrosis factor-α (TNF-α), Leukemia inhibitory factor (LIF), IFN-γ Induced Protein-10 (IP10 or CXCL10), Keratinocytes-derived chemokine (KC or CXCL1), Monocyte chemoattractant protein-1 (MCP-1 or CCL2), Monokine induced by IFN-γ (MIG or CXCL9), Macrophage inflammatory protein (MIP-1α or CCL3) and MIP-1β (or CCL4), MIP-2 (or CXCl2), RANTES (or CCL5),

Lipopolysaccharide-induced CXC chemokine (LIX or CXCL5), Eotaxin (CCL11), Granulocyte-colony stimulating factor (G-CSF), Macrophage colony-stimulating factor (M-CSF), Granulocyte-macrophage colony-stimulating factor (GM-CSF), and Vascular endothelial growth factor (VEGF).

Taqman Low Density Arrays (TLDA). Hippocampus (HC) and prefrontal cortex (PFC) samples used for the TLDA analysis were generated by the micropunch technique [5]. Briefly, coronal 200µm brain sections were prepared using a cryostat tissue slicer (Leica Biosystems, Germany). Punches (diameter: 1mm) were then excised from the HC and PFC, which were delimited according to the Franklin & Paxinos Mouse Brain Atlas (HC: between -1.46 and -3.64 mm posterior to bregma; PFC: between +2.34 and +1.54 mm posterior to bregma), and stored at −80°C. Before being reversed-transcribed to cDNA using the SuperScript™ VILO™ cDNA Synthesis Kit (Invitrogen, Thermo-Fisher Scientific, France), the total RNA extracted from these micropunches was analyzed using a 2100 Bioanalyzer (Agilent Technologies, Santa Clara, CA, USA) with an average RNA integrity number superior to 8,5 on a scale of 0 to 10. A total of 100µl reaction mixture with 50µl cDNA template (obtained from 600ng RNA) and an equal volume of TaqMan® universal master mix was added to each line of TLDA after gentle vortex mixing. Thermal cycler conditions were as follows: 2min at 50°C, 10min at 94.5°C, 30s at 97°C, and 1min at 59.7°C for 40 cycles. The threshold cycle Ct was automatically given by QuantStudio software package (Applied Biosystems, France). The cards were cycled in an OpenArray NT Cycler System (Applied Biosystems, France) at the Integrative Microgenomic platform (@BRIDGe, INRA, Jouy-en-Josas, france) following the manufacturer’s protocol. Data were extracted using the “Digital Science Thermo-Fisher Scientific” software (https://www.thermofisher.com/fr/fr/ home/digital-science.html). Detailed list of genes analyzed is provided in Table S2 (see Additional file 3).

Z-scores calculation. Z-scores were calculated as first described by Guilloux et al. (2011) [6] and show how many standard deviations a given observation is above or below the mean of the control group (here unstressed-mice fed standard-diet). The following formula was used: z-score = (the individual data for the observed parameter – mean of the control group)/ standard deviation of the control group. For emotionality z-score, z-scores were first computed from the behavioral data within each test, then across the tests for equal weighting of the different tests.

References

1. Couroussé T, Bacq A, Belzung C, Guiard B, Balasse L, Louis F, et al. Brain organic cation transporter 2 controls response and vulnerability to stress and GSK3β signaling. Mol Psychiat. 2015;20:289‑900.

2. Surget A, Saxe M, Leman S, Ibarguen-Vargas Y, Chalon S, Griebel G, et al. Drug-dependent requirement of hippocampal neurogenesis in a model of depression and of antidepressant reversal. Biol Psychiatry. 2008;64:293‑301.

3. André C, Dinel A-L, Ferreira G, Layé S, Castanon N. Diet-induced obesity progressively alters cognition, anxiety-like behavior and lipopolysaccharide-induced depressive-like behavior: focus on brain indoleamine 2,3-dioxygenase activation. Brain Behav Immun. 2014;41:10‑21.

4. Dulawa SC, Hen R. Recent advances in animal models of chronic antidepressant effects: the novelty-induced hypophagia test. Neurosci Biobehav Rev. 2005;29:771‑83.

5. Romaní-Pérez M, Lépinay AL, Alonso L, Rincel M, Xia L, Fanet H, et al. Impact of perinatal exposure to high-fat diet and stress on responses to nutritional challenges, food-motivated behaviour and mesolimbic dopamine function. Int J Obes. 2017;41:502‑9.

6. Guilloux JP, Seney M, Edgar N, Sibille E, 2011. Integrated behavioral z-scoring increases the sensitivity and reliability of behavioral phenotyping in mice: relevance to emotionality and sex. J Neurosci Methods. 2011;197:21–31.
