# Supplementary material for: A new experimental design to study inflammation-related versus non-inflammation-related depression in mice
Source: J Neuroinflammation. 2021 Dec 11;18:290. doi: 10.1186/s12974-021-02330-9 (PMC8666053; doi:10.1186/s12974-021-02330-9)
Supplement: Supplementary file 2 — Additional file 2: Table S1. Detailed daily schedule of the UCMS protocol. [file 12974_2021_2330_MOESM2_ESM.pdf]

A new experimental design to study inflammation-related versus non-inflammation-related depression in mice

Table S1: Detailed daily schedule of the unpredictable chronic mild stress (UCMS) protocol. W: week (of UCMS), h: hour, L/D: light/dark.

	Monday	Tuesday	Wednesday	Thursday	Friday	Saturday	Sunday
**W1**	No bedding : 14h50-16h20<br>New bedding	3 bedding changes 10h30-11h30<br>Social stress: 15h, 5mn	Wet bedding : 9h-11h<br>New bedding	Restraint : 12h-13h<br>Cages 45° : 14h-15h30	No bedding : 9h30-11h<br>Inversion of L/D cycle in the evening	Inversion of L/D cycle + L/D episodes every hour during 4h	Inversion of L/D cycle + L/D episodes every hour during 4h
**W2**	9h : end of L/D cycle inversion<br>Wet bedding : 12h-14h<br>New Bedding<br>Cages 45° : 14h30-16h	Aggressor bedding : 9h30-11h30<br>10 sec in water (20°C) : 15h	No bedding : 10h-11h<br>Restraint : 11h-12h<br>New Bedding	Social stress : 11h, 5mn<br>Cages 45° : 12h30-14h30<br>Social stress: 15h30, 5mn<br>Inversion of L/D cycle (20h)	No bedding : 10h30-11h30<br>Inversion of L/D cycle (end at 20h)	L/D episodes every hour during 5h	L/D episodes every hour during 5h
**W3**	Restraint : 9h30-11h30<br>Wet bedding : 12h-14h<br>New Bedding	Cages 45° : 9h-11h<br>Powerful light : 10h30-11h30<br>Cages 45° : 15h-16h	No bedding : 10h-12h<br>Social Stress : 15h, 5mn	Cages 45° : 12h-13h<br>New Bedding	Wet bedding : 12h-15h<br>Social stress : 14h30, 5mn<br>New Bedding	L/D episodes every hour during 6h	L/D episodes every hour during 6h
**W4**	Cages 45° : 10h-11h30<br>New Bedding	Agressor bedding : 10h-11h<br>Cages 45° : 13h-14h	Inversion of L/D cycle	Powerful light : 9h30-10h30<br>Cages 45° : 11h-13h	L/D episodes every hour : 12h-16h<br>Inversion of L/D cycle after 20h	Inversion of L/D cycle	Inversion of L/D cycle
**W5**	9h : end of L/D cycle inversion<br>Social Stress : 10h, 10mn<br>Restraint : 13h30-14h30	L/D episodes every h during 5h	10 s in water (20°C) : 10h<br>Water in the cage : 13h-13h30<br>New Bedding	3 bedding change : 11h-13h	Social stress : 11h, 2h<br>Restraint : 13h-14h<br>Rat bedding : 2h	L/D episodes every hour during 7h	L/D episodes every hour during 7h
**W6**	Social stress : 9h, 2h<br>Cages 45° : 12h30-14h30<br>Rat bedding : 3 h	Cages 45° : 10h-12h<br>No bedding: 13h30-15h30	Wet bedding : 10h-11h<br>New Bedding<br>Powerful light : 14h-16h	Inversion of L/D cycle<br>Restraint 2h	Cages 45° : 12h-14h<br>Social stress : 15h30, 1h<br>Rat bedding : 1 h	L/D episodes every hour during 8h	L/D episodes every hour during 8h
**W7**	Wet bedding : 13h30-15h<br>New Bedding<br>Rat bedding 2h	Cages 45° : 9h30-11h30<br>Powerful light : 11h-12h<br>No bedding : 14h-15h	No bedding : 10h-14h<br>Social Stress : 15h, 2h	10 s in water (20°C) : 11h<br>Cages 45 °C : 15h-18h	Wet bedding : 8h30-10h30<br>New bedding<br>Restraint : 12h-14h	L/D episodes every hour during 6h	L/D episodes every hour during 6h
