# Supplementary material for: A new experimental design to study inflammation-related versus non-inflammation-related depression in mice
Source: J Neuroinflammation. 2021 Dec 11;18:290. doi: 10.1186/s12974-021-02330-9 (PMC8666053; doi:10.1186/s12974-021-02330-9)
Supplement: Supplementary file 3 — Additional file 3: Table S2. List of genes analyzed in the Taqman low-density arrays (TLDA). [file 12974_2021_2330_MOESM3_ESM.pdf]

A new experimental design to study inflammation-related versus non-inflammation-related depression in mice

Table S2: List of genes analyzed in the Taqman Low Density Arrays (TLDA).

Gene	Accession number	Function
IL1b (Interleukin-1β)	Mm00434228_m1	Inflammation and microglial markers
IL6 (Interleukin-6)	Mm00446190_m1	
TNF-a (Tumor Necrosis Factor-α)	Mm00443258_m1	
IFN-g (Interferon-γ)	Mm00801778_m1	
IL-10 (Interleukin-10)	Mm00439616_m1	
IL-4 (Interleukin-4)	Mm00445259_m1	
IL-9 (Interleukin-9)	Mm00434305_m1	
TGF-β (Transforming Growth Factor-β)	Mm03024053_m1	
CCl2 - MCP1 (Monocyte Chemoattractant Protein-1)	Mm00441242_m1	
CxCl10 - IP10 (IFN-g Induced Protein-10)	Mm00445235_m1	
CxCl9 - MIG (Monokine Induced by Gamma IFN)	Mm00434946_m1	
CxCl1 - KC (Keratinocytes-derived Chemokine)	Mm00433859_m1	
Cd11b (Cluster of Differentiation 11b)	Mm00434455_m1	
Cd86 (Cluster of Differentiation 86)	Mm00444543_m1	
Cd74 (Cluster of Differentiation 74)	Mm00658576_m1	
IDO1 (Indoleamine 2,3 dioxygenase-1)	Mm00492590_m1	Kynurenine pathway
KMO (Kynurenine 3-Monoxygenase)	Mm00505511_m1	
Haao (Hydroxyanthranilic Acid Oxygenase)	Mm00517945_m1	
KAT (Kynurenine Aminotransferase)	Mm01351821_m1	
KYNU (Kynureninase)	Mm00551012_m1	
GCH1 (Guanosine Triphosphate Cyclohydrolase-1)	Mm01322973_m1	Tetrahydrobiopterin (BH4) pathway
GFRP (GTP cyclohydrolase-1Feedback Regulator)	Mm00622819_m1	
SPR (Sepiapterin Reductase)	Mm00488430_m1	
DHFR (Dihydrofolate Reductase)	Mm00515662_m1	
PTPS (6-Pyruvoyl-tetrahydropterin Synthase)	Mm00478494_m1	
5-HT1aR (Serotonin 1a Receptor)	Mm00434106_s1	5-HT system
5-HT1bR (Serotonin 1b Receptor)	Mm00439377_s1	
5-HT2cR (Serotonin 2c Receptor)	Mm00434127_m1	
Slc18a1-VMAT2 (Vesicular Monoamine Transporter 2)	Mm00461868_m1	
Slc6a4 - 5HTT (Serotonin Transporter)	Mm00439391_m1	

Maoa (Monoamine oxidase a)	Mm00558004_m1	
Maob (Monoamine oxidase b)	Mm00555412_m1	
Slc6a2-NET (Norepinephrine (NE) Transporter)	Mm00436661_m1	NE system
Slc17a7-vGlut (Vesicular Glutamate Transporter)	Mm00812886_m1	Glutamate system
Slc1a2-GLT1 (Glutamate Transporter)	Mm00441457_m1	
Slc1a3-Glast (Glial High Affinity Glutamate Transporter)	Mm00600697_m1	
Grin2a-NR2a (Glutamate Receptor NMDA subunit 2a)	Mm00433803_m1	
Grin2b-NR2b (Glutamate Receptor NMDA subunit 2b)	Mm01265044_m1	
Nos1 (Nitric Oxide synthase 1)	Mm01208059_m1	Oxidative status
Nos2 (Nitric Oxide synthase 2)	Mm00440485_m1	
CAT (Catalase)	Mm00437992_m1	
GPx-1 (Glutathione Peroxidase-1)	Mm00656767_g1	
SOD (Superoxide dismutase-1)	Mm01700393_g1	
GFAP (Glial Fibrillary Acidic Protein)	Mm01253033_m1	Astrocyte marker
GAPDH (Glyceraldehyde Phosphate Dehydrogenase)	Mm99999915_g1	Housekeeping genes
B2M (β2-Microglobulin)	Mm00437762_m1	
Actin β	Mm00607939_s1	
18S (ribosomal protein S18)	Mm04277571_s1	
