# Supplementary material for: A new experimental design to study inflammation-related versus non-inflammation-related depression in mice
Source: J Neuroinflammation. 2021 Dec 11;18:290. doi: 10.1186/s12974-021-02330-9 (PMC8666053; doi:10.1186/s12974-021-02330-9)
Supplement: Supplementary file 4 — Additional file 4: Table S3. Plasma levels of adipokines, corticosterone and glucose measured at the end of the experiment. [file 12974_2021_2330_MOESM4_ESM.pdf]

A new experimental design to study inflammation-related versus non-inflammation-related depression in mice

Table S3: **Plasma levels of adipokines, corticosterone and glucose measured at the end of the experiment.**

	Controls	UCMS	HFD	HFD-UCMS
Leptin (ng/ml)	2.97 ± 0.89	3.12 ± 0.51	15.88 ± 0.85***	16.75 ± 0.80***
Resistin (ng/ml)	22.05 ± 3.55	23.55 ± 2.76	33.90 ± 3.61***	37.09 ± 4.19***
Adiponectin (mg/ml)	11.60 ± 1.51	13.88 ± 1.31	13.13 ± 1.67	13.51 ± 2.12
Corticosterone (ng/ml)	8.99 ± 2.35	19.15 ± 8.48§	12.41 ± 1.82	22.29 ± 4.32§
Fasted glycemia (mg/dl)	109.67 ± 3.99	128.50 ± 5.34###	152.80 ± 6.47***	175.57 ± 7.42*** ###

n=10/group for corticosterone; n=14-16/group for the other analytes. ***P<0.001 for HFD effect; §P=0.06, ###p<0.001 for Stress effect.
