# Supplementary material for: A new experimental design to study inflammation-related versus non-inflammation-related depression in mice
Source: J Neuroinflammation. 2021 Dec 11;18:290. doi: 10.1186/s12974-021-02330-9 (PMC8666053; doi:10.1186/s12974-021-02330-9)
Supplement: Supplementary file 5 — Additional file 5: Fig. S1. HFD mice displayed expected increase of circulating concentrations of inflammatory factors. Plasma levels of cytokines and chemokines analyzed at the end of the experiment in unstressed (Controls) or stressed (UCMS) SD and HFD mice. Detailed statistical analysis reported significant differences between groups for circulating levels of: (A) IL-6, (B) TNF-α, (C) IL-3, (D) IL-5, (E) IL-10, (F) IL-13, (G) MIG or CXCL9, (H) IP10 or CXCL10, (I) KC or CXCL1, (J) MCP-1 or CCL2, (K) MIP-1β or CCL4, (L) G-CSF and (M) CCL11. n = 8–15 mice/group. All results are graphed as means ± SEM. **P < 0.01, ***P < 0.001 for Diet effect; #P < 0.05 for Stress effect; $P < 0.05, $$P < 0.01, $$$P < 0.001 for differences vs. unstressed-SD mice; ++P < 0.01, +++P < 0.001 for differences vs. unstressed-HFD mice. [file 12974_2021_2330_MOESM5_ESM.pdf]

A new experimental design to study inflammation-related versus non-inflammation-related depression in mice

Fig. S1: HFD mice displayed expected increase of circulating concentrations of inflammatory factors.

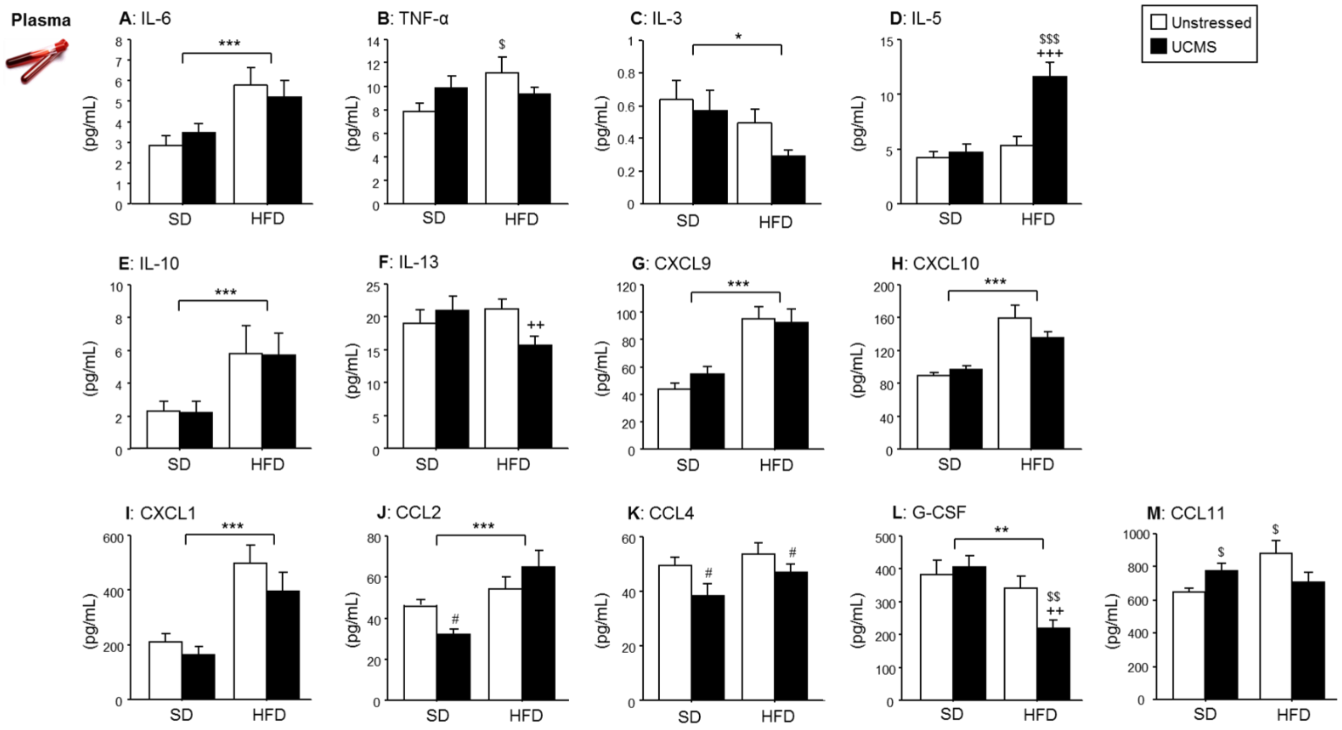

Fig. S1: HFD mice displayed expected increase of circulating concentrations of inflammatory factors. Plasma levels of cytokines and chemokines analyzed at the end of the experiment in unstressed (Controls) or stressed (UCMS) SD and HFD mice. Detailed statistical analysis reported significant differences between groups for circulating levels of: (**A**) interleukin-6 (IL-6), (**B**) tumor necrosis factor-α (TNF-α), (**C**) IL-3, (**D**) IL-5, (**E**) IL-10, (**F**) IL-13, (**G**) monokine induced by IFN-γ (MIG or CXCL9), (**H**) IFN-γ induced protein-10 (IP10 or CXCL10), (**I**) keratinocytes-derived chemokine (KC or CXCL1), (**J**) monocyte chemoattractant protein-1 (MCP-1 or CCL2), (**K**) macrophage inflammatory protein-1β (MIP-1β or CCL4), (**L**) granulocyte-colony stimulating factor (G-CSF) and (**M**) Eotaxin (CCL11). n=8-15 mice/group. All results are graphed as means ± SEM. **P<0.01, ***P<0.001 for Diet effect; $^{\#}$P<0.05 for Stress effect; $^{\$}$P<0.05, $^{\$\$}$P<0.01, $^{\$\$\$}$P<0.001 for differences *vs.* unstressed-SD mice; $^{++}$P<0.01, $^{+++}$P<0.001 for differences *vs.* unstressed-HFD mice.
