# Supplementary material for: A new experimental design to study inflammation-related versus non-inflammation-related depression in mice
Source: J Neuroinflammation. 2021 Dec 11;18:290. doi: 10.1186/s12974-021-02330-9 (PMC8666053; doi:10.1186/s12974-021-02330-9)
Supplement: Supplementary file 6 — Additional file 6: Fig. S2. HFD and UCMS differentially modulated HC gene expression of inflammatory markers and related neurobiological processes. Relative gene expression (as compared to controls) measured by TLDA analysis in the hippocampus (HC) of unstressed (Controls) or stressed (UCMS) SD and HFD mice. Detailed analysis revealed significant impact of HFD and/or UCMS for: (A) inflammatory cytokines and markers of microglial activation (IL-1β, IL-6, CD11b, CD74); (B) enzymes from the kynurenine (KYN) pathway (KAT, KYNU); (C) enzymes from the tetrahydrobiopterin (BH4) pathway (GCH1, GFRP, SPR, DHFR, PTPS); (D) key elements of the 5-HT system (5-HT1A, 5-HT1B, 5-HT2C receptors, 5-HT transporter (5-HTT), monoamine oxidase A (MAOA)); (E) markers of glutamate system (vGLUT, GLT-1, GLAST) and (F) oxidative enzymes (NOS2, CAT). (n = 8–10 mice/group). All results are graphed as means ± SEM. *P < 0.05, **P < 0.01 for Diet effect; #P < 0.05, ##P < 0.01 for Stress effect; $P < 0.05, $$P < 0.01 for differences vs. unstressed-SD mice; +P ≤ 0.05 for differences vs. unstressed-HFD mice. [file 12974_2021_2330_MOESM6_ESM.pdf]

A new experimental design to study inflammation-related versus non-inflammation-related depression in mice

Fig. S2: HFD and UCMS differentially modulated hippocampus gene expression of inflammatory markers and related neurobiological processes.

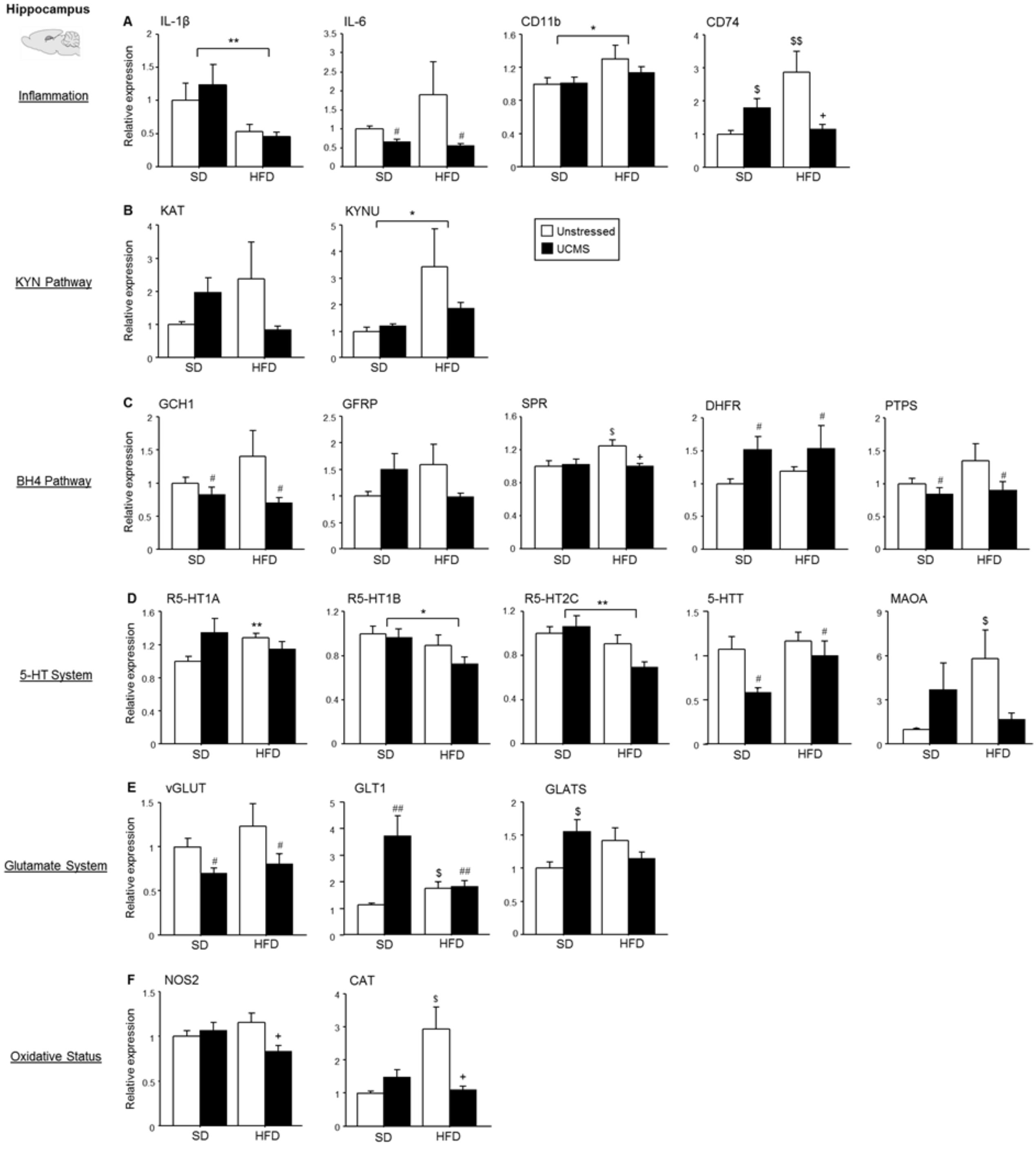

Fig. S2: HFD and UCMS differentially modulated hippocampus (HC) gene expression of inflammatory markers and related neurobiological processes. Relative gene expression (as compared to controls) measured by TLDA analysis in unstressed (Controls) or stressed (UCMS) SD and HFD mice. Detailed analysis revealed significant impact of HFD and/or UCMS for: (**A**) inflammatory cytokines and markers of microglial activation (*IL-1β, IL-6*, *CD11b*, *CD74*); (**B**) enzymes from the kynurenine (KYN) pathway (*KAT, KYNU*); (**C**) enzymes from the tetrahydrobiopterin (BH4) pathway (*GCH1*, *GFRP*, *SPR*, *DHFR*, *PTPS*); (**D**) key elements of the 5-HT system (*5-HT1A*, *5-HT1B*, *5-HT2C receptors*, *5-HTT*, *MAOA*); (**E**) markers of glutamate system (*vGLUT*, *GLT-1*, *GLAST*) and (**F**) oxidative enzymes (*NOS2*, *CAT*). (n=8-10 mice/group). All results are graphed as means ± SEM. *P<0.05, **P<0.01 for Diet effect; $^{\#}$P<0.05, $^{\#\#}$P<0.01 for Stress effect; $^{\$}$P<0.05, $^{\$\$}$P<0.01 for differences *vs.* unstressed-SD mice; $^{+}$P≤0.05 for differences *vs.* unstressed-HFD mice.

Supplementary Figure S3

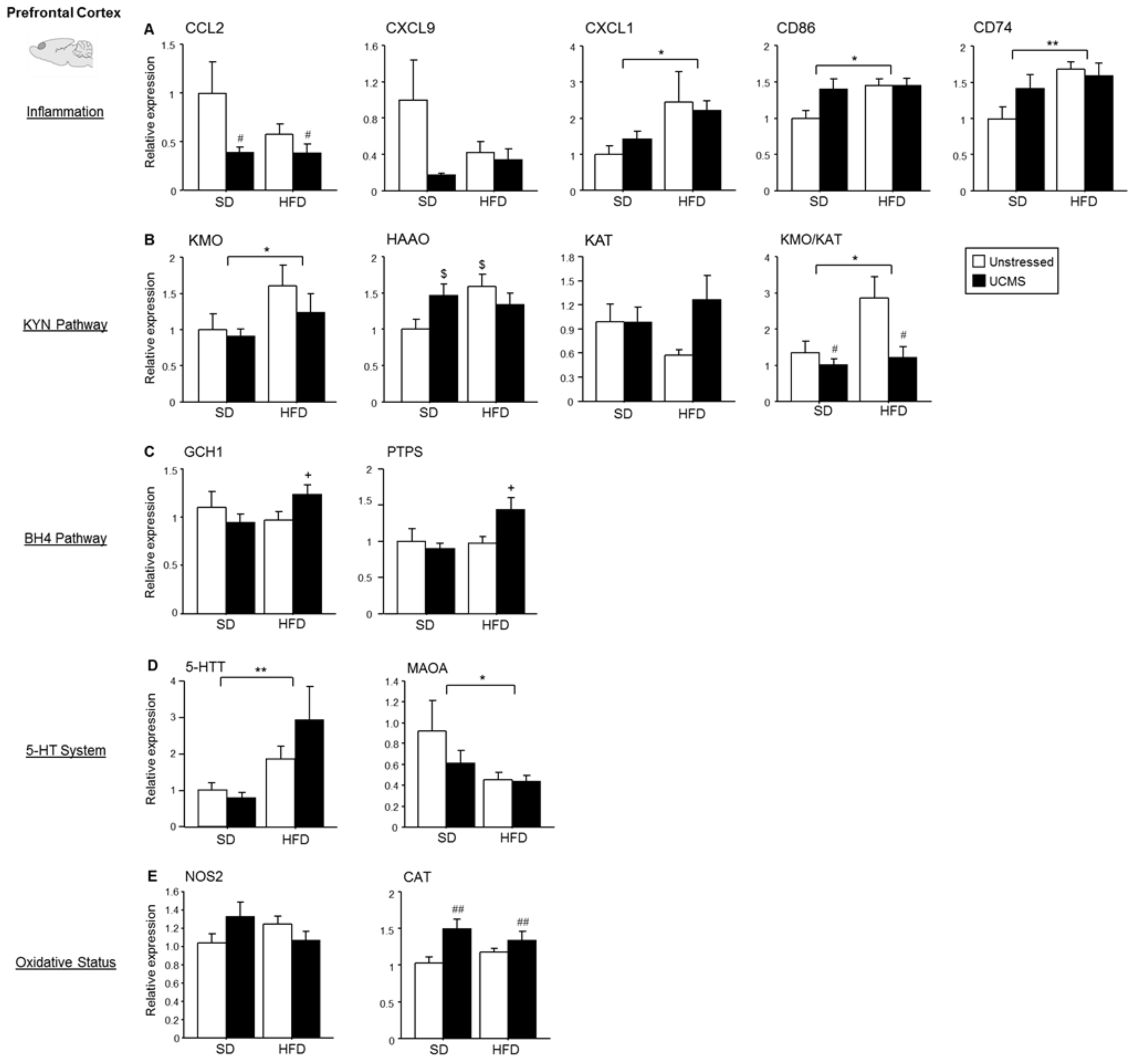

Supplementary Figure S3: HFD and UCMS differentially modulated prefrontal cortex (PFC) gene expression of inflammatory markers and related neurobiological processes. Relative gene expression (as compared to controls) measured by TLDA analysis in unstressed (Controls) or stressed (UCMS) SD and HFD mice. Detailed analysis revealed significant impact of HFD and/or UCMS for: (**A**) Markers of microglial activation (*CCL2, CXCL9, CXCL1, CD86* and *CD74*); (**B**) enzymes from the KYN pathway (*kynurenine 3-monoxygenase* (KMO), *hydroxyanthranillic acid oxygenase* (HAAO), KAT) and the neurotoxicity/neuroprotection

ratio (expression level of KMO/KAT); (**C**) enzymes from the BH4 pathway (GCH1, PTPS); (**D**) key elements of the 5-HT system (5-HTT, MAOA) and (**E**) oxidative enzymes (NOS2, CAT). (n=8-10 mice/group). All results are graphed as means ± SEM. *P<0.05, **P<0.01 for Diet effect; $^{\#}$P<0.05 for Stress effect; $^{\$}$P<0.05 for differences *vs.* unstressed-SD mice; $^{+}$P<0.05 for differences *vs.* unstressed-SD mice.
