# Supplementary material for: A new experimental design to study inflammation-related versus non-inflammation-related depression in mice
Source: J Neuroinflammation. 2021 Dec 11;18:290. doi: 10.1186/s12974-021-02330-9 (PMC8666053; doi:10.1186/s12974-021-02330-9)
Supplement: Supplementary file 7 — Additional file 7: Fig. S3. HFD and UCMS differentially modulated PFC gene expression of inflammatory markers and related neurobiological processes. Relative gene expression (as compared to controls) measured by TLDA analysis in the prefrontal cortex (PFC) of unstressed (Controls) or stressed (UCMS) SD and HFD mice. Detailed analysis revealed significant impact of HFD and/or UCMS for: (A) Markers of microglial activation (CCL2, CXCL9, CXCL1, CD86, CD74); (B) enzymes from the KYN pathway (KMO, HAAO, KAT) and the neurotoxicity/neuroprotection ratio (expression level of KMO/KAT); (C) enzymes from the BH4 pathway (GCH1, PTPS); (D) key elements of the 5-HT system (5-HTT, MAOA) and (E) oxidative enzymes (NOS2, CAT). (n = 8–10 mice/group). All results are graphed as means ± SEM. *P < 0.05, **P < 0.01 for Diet effect; #P < 0.05 for Stress effect; $P < 0.05 for differences vs. unstressed-SD mice; +P < 0.05 for differences vs. unstressed-SD mice. [file 12974_2021_2330_MOESM7_ESM.pdf]

A new experimental design to study inflammation-related versus non-inflammation-related depression in mice

Fig. S3: HFD and UCMS differentially modulated PFC gene expression of inflammatory markers and related neurobiological processes.

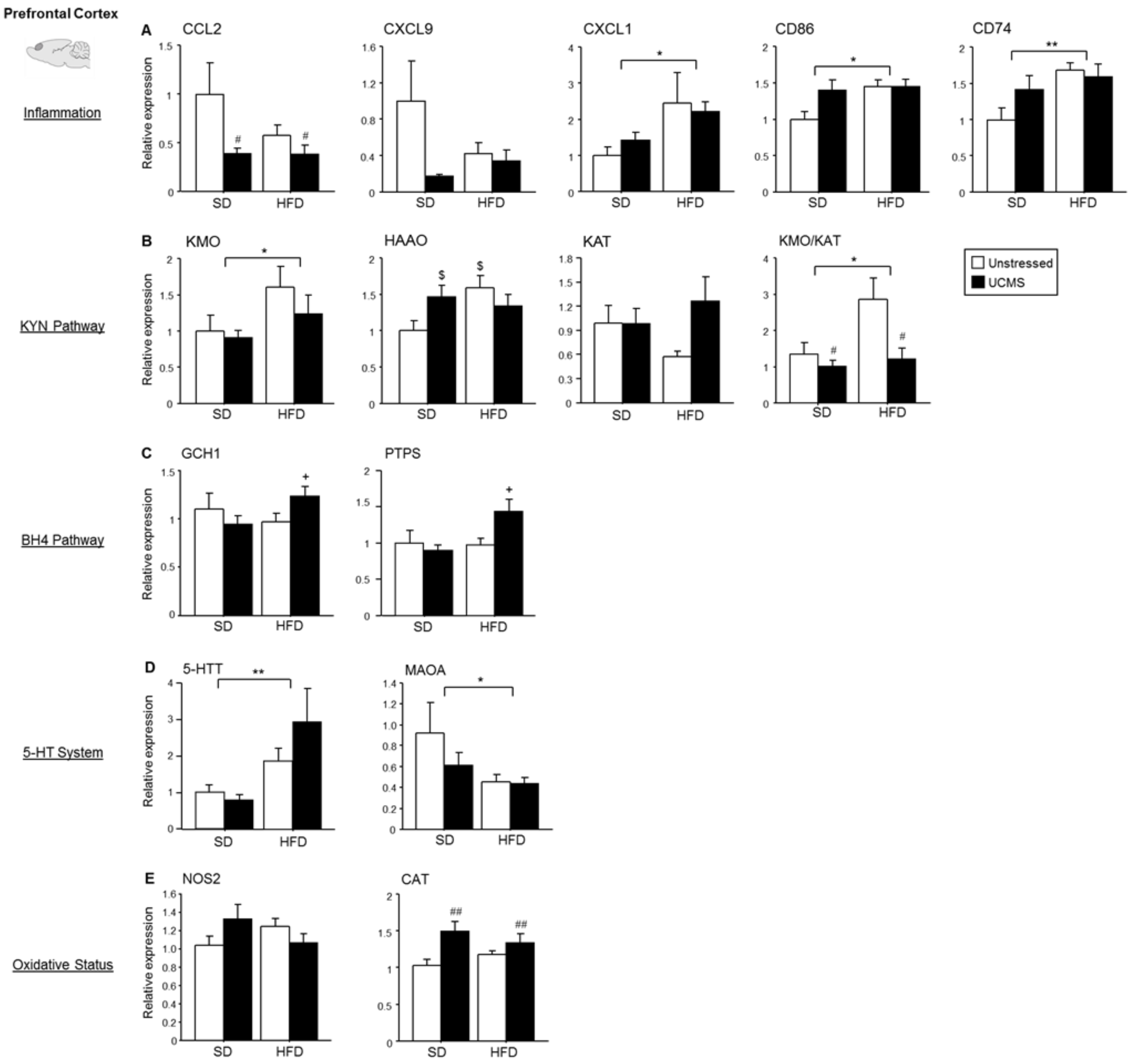

Fig. S3: HFD and UCMS differentially modulated PFC gene expression of inflammatory markers and related neurobiological processes. Relative gene expression (as compared to controls) measured by TLDA analysis in the prefrontal cortex (PFC) of unstressed (Controls) or stressed (UCMS) SD and HFD mice. Detailed analysis revealed significant impact of HFD and/or UCMS for: (**A**) Markers of microglial activation (*CCL2*, *CXCL9*, *CXCL1*, *CD86* and

CD74); (**B**) enzymes from the KYN pathway (*KMO*, *HAAO*, *KAT*) and the neurotoxicity/neuroprotection ratio (expression level of *KMO/KAT*); (**C**) enzymes from the BH4 pathway (*GCH1*, *PTPS*); (**D**) key elements of the 5-HT system (*5-HTT*, *MAOA*) and (**E**) oxidative enzymes (*NOS2*, *CAT*). (n=8-10 mice/group). All results are graphed as means ± SEM. *P<0.05, **P<0.01 for Diet effect; $^{\#}$P<0.05 for Stress effect; $^{\$}$P<0.05 for differences *vs.* unstressed-SD mice; $^{+}$P<0.05 for differences *vs.* unstressed-SD mice.
